# Supplementary material for: AutoCoEv—A High-Throughput In Silico Pipeline for Predicting Inter-Protein Coevolution
Source: Int J Mol Sci. 2022 Mar 20;23(6):3351. doi: 10.3390/ijms23063351 (PMC8952222; doi:10.3390/ijms23063351)
Supplement: Supplementary file 1 [file ijms-23-03351-s001.zip › ijms-1646085-supplementary.pdf]

International Journal of
Molecular Sciences

MDPI

Supplementary

AutoCoEv – a high-throughput *in silico* pipeline for predicting inter-protein coevolution

Petar B. Petrov [1,2,*], Luqman O. Awoniyi [1,2], Vid Šuštar [2], M. Özge Balci [1,2] and Pieta K. Mattila [1,2,*]

1 Institute of Biomedicine and MediCity Research Laboratories, University of Turku, Turku, Finland
2 Turku Bioscience, University of Turku and Åbo Akademi University, Turku, Finland
* Correspondence: petar.petrov@utu.fi (PBP); pieta.mattila@utu.fi (PKM)

Supplementary figures

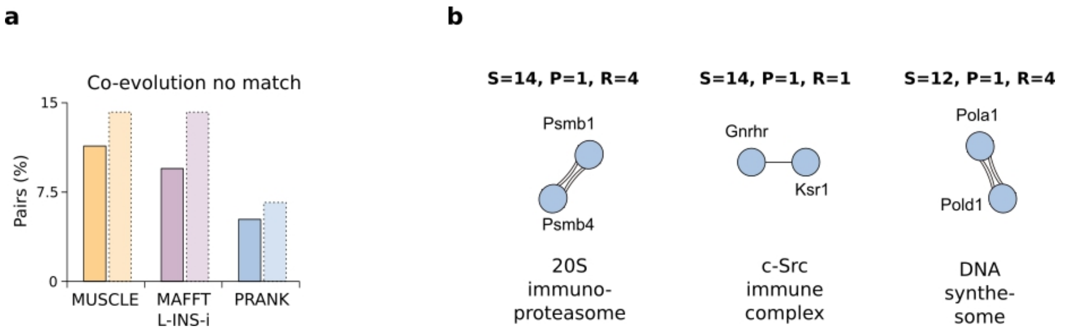

Supplementary Figure S1. Determining AutoCoEv strategy. **A)** Non-matched coevolution. Protein pairs for which coevolution was detected in both CAPS2 runs, but not on the same amino acids. **B)** CORUM database. Coevolution was detected in 5 out of the 10 largest complexes. The first 2 are shown in Figure 2B from the main text; shown here are the second 3.

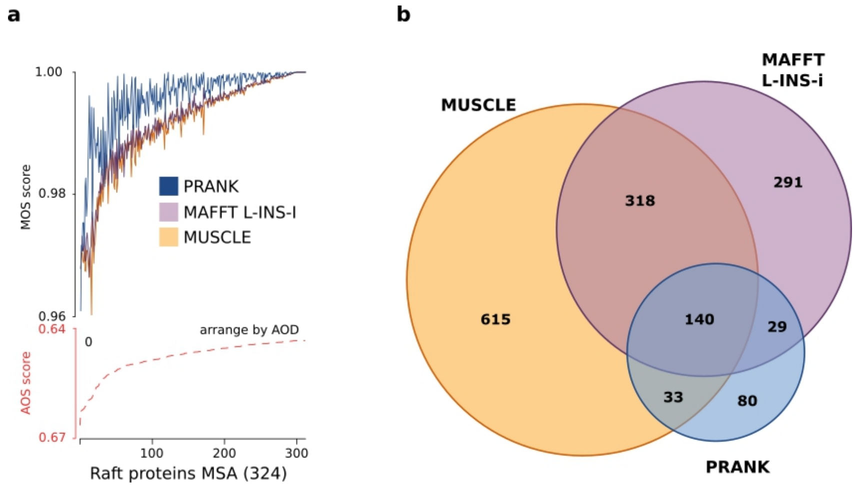

Supplementary Figure S2. Assessing the alignment methods for the large-scale protein dataset of 324 lipid-rafts proteins. **A)** The quality of the MSAs. Alignments created by MUSCLE, MAFFT L-INS-i and PRANK were compared by Mumsa and sorted by their average overlap score (AOS), which reflects the difficulty of aligning the sequences. The multiple overlap score (MOS), which indicates the quality of each individual alignment was plotted for the three methods. **B)** Comparison of protein pairs detected to coevolve. Overlap of the protein pairs for which coevolution was detected from alignments created by MUSCLE, MAFFT L-INS-i and PRANK is indicated in absolute numbers.

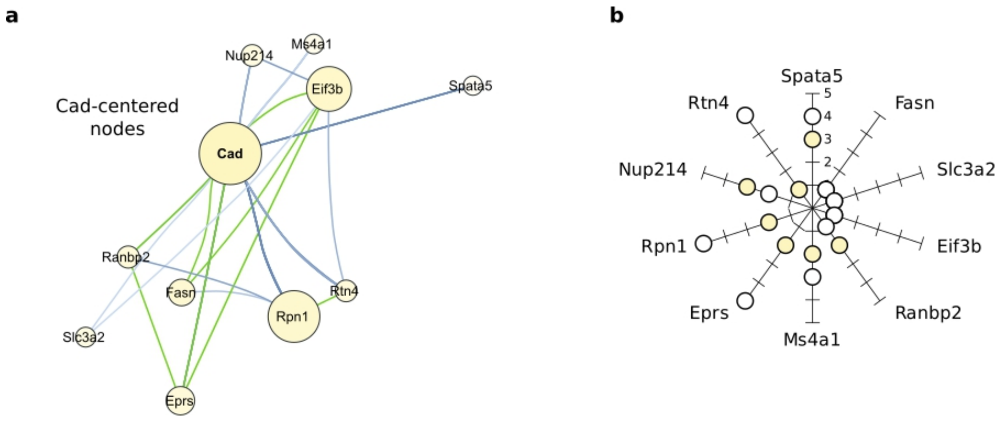

Supplementary Figure S3. Proteins predicted to coevolve with Cad. **A)** A cluster formed by proteins predicted to coevolve with Cad. Protein interactions supported also by STRING are shown in green. **B)** Number of coevolving sites from Cad and from its interacting proteins. The numbers of coevolving sites from Cad (yellow circle) and each of the proteins predicted to coevolve with it (white circle) are indicated in the schematic on the scale of 1-5 for each protein pair. For example, there are 4 sites from Spata5 coevolving with 3 sites from Cad.

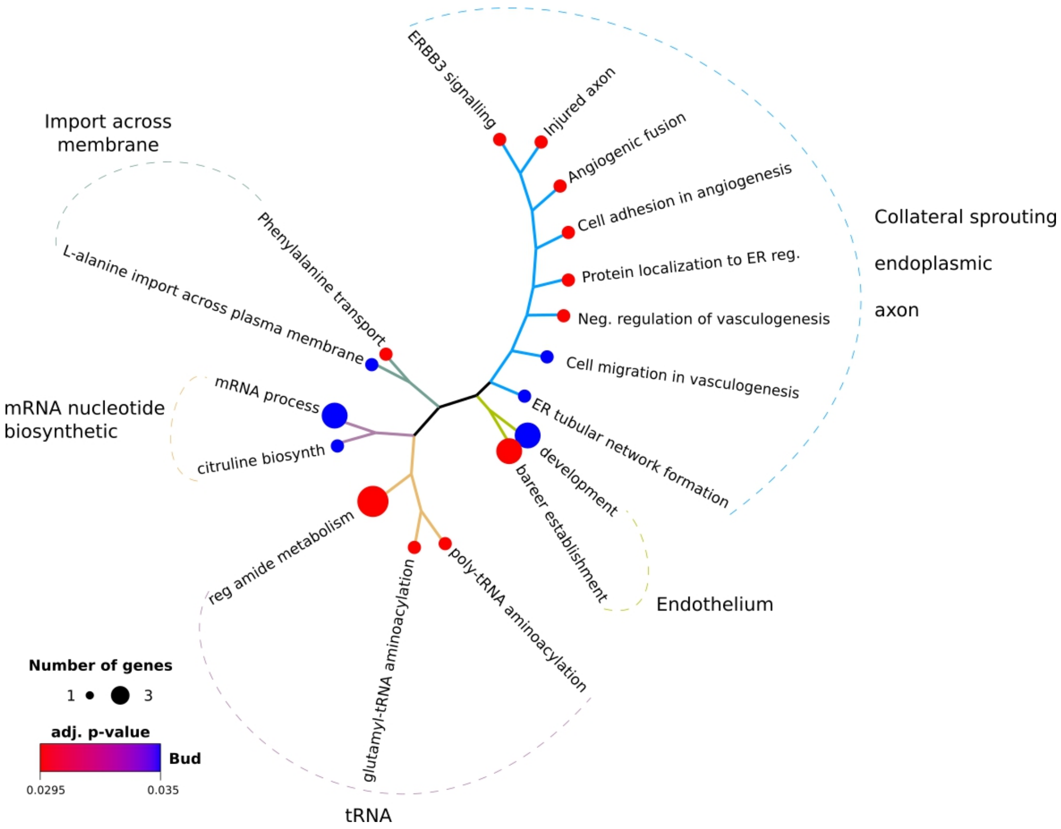

Supplementary Figure S4. Gene ontology analysis of the clustered proteins. The cellular processes, in which the proteins are involved, are indicated.

Supplementary information

P-values of the results

First, based on the input data, CAPS2 simulates a number (e.g. 100) of random MSA pairs, and tests all sites combinations for coevolutionary correlation. The acquired correlation values are stored in a vector, the size of which (V_{SIZE}) depends on the MSA lengths of the two proteins (L_1 and L_2), and the number of simulations (r) performed (Equation 1). The correlations are sorted by value (from 0 to 1) and the vector hereafter serves as a null data distribution to which the real data will be compared. The α-value derives an index number (I_{THRESH}) within the vector (Equation 2), whereas the value contained at the indexed position is set as a correlation threshold (C_{THRESH}) (Figure S1).

(1) $V_{SIZE} = L_1 \times L_2 \times r$

(2) $I_{THRESH} = V_{SIZE}(1 - \alpha) + 1$

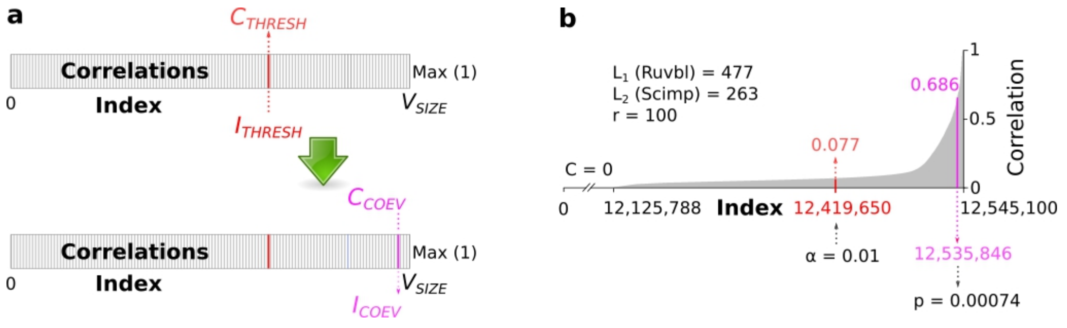

SI Figure S1. Determining p-values. A) Threshold and correlation. Correlation values of simulated data are stored in a vector (rectangular box) and sorted by size from 0 to 1 (Max). The vector size (V_{SIZE}) reflects the total number of possible correlations multiplied by the number of simulations. The threshold position within the vector (index, I_{THRESH}) is determined by α in Equation 1, and its correlation value (C_{THRESH}) is set as a cut-off for the correlations detected in the user-provided data (C_{COEV}). With our patch, CAPS2 ranks the position of each detected correlation value within the vector, determines its index (I_{COEV}) and uses it in Equation 2 to calculate the corresponding p-value. B) Example data. Protein pair Ruvb1 and Scimp was analysed in our preliminary runs. The lengths of Ruvb1 and Scimp MSAs (L1 and L2) and the number of simulations (r) are reflected in V_{SIZE} = 12,545,100. The contents of the vector holding correlation values (C) from the simulated data are shown: C = 0 (no correlation) in blue (X-axis) and C > 0 in grey, starting at index 12,125,788. The threshold value position (red) is determined by α in Equation 1 and returns C_{THRESH} = 0.077. A pair of coevolving with a C_{COEV} = 0.686, was ranked within the vector at index 12,535,846; the index was in turn used in Equation 3 to calculate p = 0.00074 for the correlation.

The threshold is thus specific for each protein pair, and correlations detected from the real data, that rank above it are deemed as being significant. With our patch (see "Patch for verbose output" next), after coevolution has been detected between two sites, CAPS2 ranks its value within the vector and determines the corresponding vector index (I_{COEV}) at that position. After "searching back" the I_{COEV} (SI Figure 1a), CAPS2 can calculate the corresponding p-value (Equation 3). An actual example is shown in SI Figure 1b.

(3) $p = \frac{(1 + V_{SIZE} - I_{COEV})}{V_{SIZE}}$

When analysing a protein pair, CAPS2 estimates the correlation between two sites bidirectionally (e.g. for sites A and B, correlation is estimated: A → B and A ← B),

exporting their mean value in the results (this is not to be confused with the bidirectional run of CAPS2 that AutoCoEv performs, by loading the two MSAs in alternating order.). On rare occasions, CAPS2 assigns a negative value at the bidirectional correlation estimation step. Since this usually yields a mean value lower than the threshold, AutoCoEv dismisses site pairs where a negative correlation was estimated.

Patch for verbose CAPS2 output

Our caps_verbose.patch introduces two modifications to CAPS2, making the program produce more verbose output. First, the program will use *TreeTemplateTools* from bpp-phyl to export CAPS2 generated trees by *treeToParenthesis*, if none were supplied by the user at run-time. When phylogenetic trees are not supplied by the user, CAPS generates its own trees automatically (Box 1).

Box S1.

```
tree1 = create_input_tree(vec1.names, vec1.sequences);
tree2 = create_input_tree(vec2.names, vec2.sequences);
```

Our patch simply outputs the trees by *treeToParenthesis* (Box 2).

Box S2.

```
string temptre1 = TreeTemplateTools::treeToParenthesis(*tree1, true);
string temptre2 = TreeTemplateTools::treeToParenthesis(*tree2, true);
```

Output of the p-values

CAPS simulates number (-r) of random MSA, where each simulated alignment has the same number of columns (length) as the real data and is tested by the same method. This ensures that the data is compared to a *null* distribution without coevolution pressures. Correlation information from the simulated data is stored and sorted into vector *totaltemp*. The correlation *threshold* for each protein pair is based on the simulated data in *totaltemp*, found at a certain index, *value*. It is determined by the alpha (-a) run-time option, referred here as *threshval* (Box 3).

Box S3.

```
[1] int value = floor(((totaltemp.size())*(1-(threshval))))+1;
threshold = totaltemp[value];
```

Correlation between residues R1 and R2 is determined as the mean of correlation R1→R2 and correlation R2→R1. Each must be higher than the *threshold*, whereas *thresholdR* is always 0.01 (Box 4).

Box S4.

```
if((fabs(Correl1[cor])>=threshold && fabs(Correl1[cor])>=thresholdR)
&& (fabs(Correl2[cor])>=threshold && fabs(Correl2[cor])>=thresholdR))
```

After coevolution between sites has been determined, CAPS2 ranks the correlation value within totaltemp vector containing the null simulated data. We seek to calculate the p-value of each correlation using the formula from [1], where *value* is the correlation closest *index* within the *totaltemp* vector. We introduce a new function *getIndex*, which ranks a value *K* within a vector *v*, defines its lower bound value as *it*, then determines the *index* of *it* within the vector. Here, *v* = *totaltemp* and *K* = Correl_[cor] and the p-value of Correl_[cor] is returned as *alphathresh* (Box 5).

Box S5.

```
double getIndex(std::vector<double> const& v, double K)
{
    auto const it = std::lower_bound(v.begin(), v.end(), fabs(K));
    if (it != v.end()) {
        int index = distance(v.begin(), it);
        alphathresh = (((int)1+(double)v.size()-(int)index)/(double)v.size());
        return alphathresh;
      }
      else {
        cerr << "ELEMENT NOT FOUND!" << endl;
    }
}
```

We use the index of lower bound *it*, since an exact match of the correlation value Correl_[cor] is unlikely to be found within *totaltemp* (Box 6).

Box S6.

```
double Pvalue1 = getIndex(totaltemp, Correl1[cor]);
double Pvalue2 = getIndex(totaltemp, Correl2[cor]);
```

Chi squared test

We attempted to replicate in AutoCoEv the χ^2 test performed by CAPS2 when more than 2 proteins are evaluated. For each single protein (hereby referred as Protein *0*) the number of coevolving amino acid pairs detected with each other protein are considered (SI Figure 2), as follows:

x: number of coevolving amino acid pairs between Protein *0* and Protein *i*
t: total number of possible amino acid pairs combinations (eq. 4) between Protein 0 and Protein *i*

(4) $t_i = L_0 \times L_1$, see eq. 1

b: background coevolution for Protein 0 against all other proteins

(5) $b = \frac{\sum_{i=1}^{n} \frac{x_i}{t_i}}{n}$

e: expected number of coevolving pairs for Protein 0 and Protein i

(6) $e_i = b t_i$

Coevolution between Protein 0 and Protein *i* passes the test if:

(7) $\chi^2 = \frac{(x_i - e_i)^2}{e_i}$

$x_i > e_i$
$x_i > 3.84146$ ($\chi^2 = 0.05$)

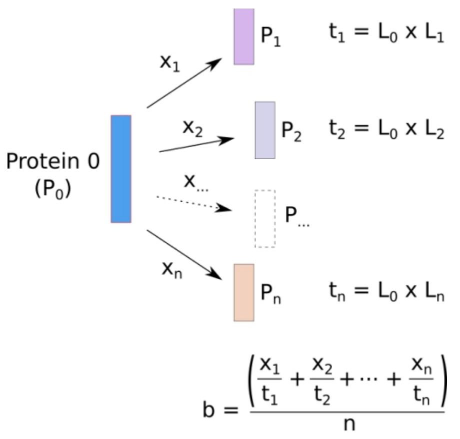

SI Figure S2. Coevolution between Protein 0 and all other proteins (n). Number of coevolving amino acid pairs (x), total number of possible amino acid combinations (t) between P_0 and each of the other proteins (P_1, P_2, ..., P_n) are indicated. Equation shows calculation of background (b), following eq. 5.

Correlation normalized to threshold

Since correlation values (C_{COEV}) derived by CAPS2 cannot be directly compared between protein pairs, AutoCoEv calculates normalized values to the threshold (C_{THRESH}) of each protein pair, where 1 is the maximum possible correlation value:

(8) $C_{NORM} = \frac{(C-T)}{(1-T)}$

Supplementary tables

Table S1. Mammalian species used for AutoCoEv analysis.

Species 1-17		Species 18-34		Species 35-50	
**taxid**	**Latin name**	**taxid**	**Latin name**	**taxid**	**Latin name**
9606	Homo_sapiens	10116	Rattus_norvegicus	9796	Equus_caballus
9598	Pan_troglodytes	10029	Cricetulus_griseus	9807	Ceratotherium_simum
9597	Pan_paniscus	10181	Heterocephalus_glaber	9615	Canis_lupus
9593	Gorilla_gorilla	10141	Cavia_porcellus	9669	Mustela_putorius
9601	Pongo_abelii	43179	Ictidomys_tridecemlineatus	9646	Ailuropoda_melanoleuca
61853	Nomascus_leucogenys	9986	Oryctolagus_cuniculus	9685	Felis_catus
60711	Chlorocebus_sabaeus	9978	Ochotona_princeps	132908	Pteropus_vampyrus
9541	Macaca_fascicularis	482537	Galeopterus_variegatus	59463	Myotis_lucifugus
9544	Macaca_mulatta	9395	Tupaia_glis	143292	Manis_pentadactyla
9555	Papio_anubis	9365	Erinaceus_europaeus	9783	Elephas_maximus
43780	Nasalis_larvatus	42254	Sorex_araneus	9785	Loxodonta_africana
61622	Rhinopithecus_roxellana	9823	Sus_scrofa	99490	Loxodonta_cyclotis
27679	Saimiri_boliviensis	9913	Bos_taurus	9813	Procavia_capensis
1868482	Carlito_syrichta	9940	Ovis_aries	9371	Echinops_telfairi
30608	Microcebus_murinus	9739	Tursiops_truncatus	9361	Dasypus_novemcinctus
30611	Otolemur_garnettii	9767	Balaenoptera_acutorostrata	9358	Choloepus_hoffmanni
10090	Mus_musculus	30538	Vicugna_pacos		

Table S2. Negatome database protein pairs from mouse (*Mus musculus*)

Prot. A	Prot. B	PubMed ID	Evidence
O08599	P70452	16186111	MI:0007 - anti tag coimmunoprecipitation
O08599	Q80T23	16186111	MI:0007 - anti tag coimmunoprecipitation
O08599	Q8K1E0	16186111	MI:0007 - anti tag coimmunoprecipitation
O08599	Q99N48	16186111	MI:0007 - anti tag coimmunoprecipitation
O08599	Q99N50	16186111	MI:0007 - anti tag coimmunoprecipitation
O08674	O35516	9111338	MI:0059 - gst pull down
O08674	P31695	9111338	MI:0059 - gst pull down
O08674	Q01705	9111338	MI:0059 - gst pull down
O08674	Q61982	9111338	MI:0059 - gst pull down
O08715	P31324	11742813	MI:1200 - immunocytochemistry
O08849	Q9Z0G0	9770488	MI:0018 - two hybrid
O08915	P53762	9083006	MI:0019 - coimmunoprecipitation
O08983	Q99KG7	12663659	MI:0018 - two hybrid
O35551	Q3UMR0	16525121	MI:0019 - coimmunoprecipitation
O35625	P70339	10428961	MI:0007 - anti tag coimmunoprecipitation
O35657	P01132	2690595	MI:0004 - affinity chromatography technology, MI:0110 - text mining
O35717	P05532	14707129	MI:0019 - coimmunoprecipitation
O35718	P05532	14707129	MI:0019 - coimmunoprecipitation
O35718	Q62120	10534114	MI:0019 - coimmunoprecipitation, MI:0110 - text mining
O35984	Q7TPV4	17875935	MI:0019 - coimmunoprecipitation
O55003	Q99JI6	17928295	MI:0018 - two hybrid
O55131	P42208	16914559	MI:0055 - fluorescent resonance energy transfer
O70355	P09793	17237401	MI:0054 - fluorescence-activated cell sorting
O70355	P31041	17237401	MI:0054 - fluorescence-activated cell sorting
O70355	Q7TSA3	17237401	MI:0054 - fluorescence-activated cell sorting
O70355	Q9WVS0	17237401	MI:0054 - fluorescence-activated cell sorting
O70433	Q62219	16737959	MI:0019 - coimmunoprecipitation
O70622	Q7M6Z0	17189258	MI:0415 - enzymatic studies
O70622	Q8K0S5	17189258	MI:0415 - enzymatic studies
O88346	P0C605	10671526	MI:0018 - two hybrid
P01582	Q61730	9620655	MI:0030 - cross-linking study, MI:0110 - text mining
P02340	Q6I6G8	12890487	MI:0019 - coimmunoprecipitation
P03995	P05627	15576676	MI:0096 - pull down; MI:0019
P05627	P28738	15576676	MI:0096 - pull down; MI:0019
P05627	P33175	15576676	MI:0096 - pull down; MI:0019
P05627	P57776	15576676	MI:0096 - pull down; MI:0019
P05627	Q61768	15576676	MI:0096 - pull down; MI:0019
P05627	Q6PER3	15576676	MI:0096 - pull down; MI:0019
P05627	Q9ESN9	15576676	MI:0096 - pull down; MI:0019
P06876	P02340	8616838	MI:0019 - coimmunoprecipitation, MI:0070 - mobility shift
P08752	Q76MZ3	15525651	MI:0059 - gst pull down
P09803	Q8BN78	10207085	MI:0019 - coimmunoprecipitation
P13405	Q61501	7675450	MI:0413 - electrophoretic mobility shift assay

P13808	Q02357	8798415	MI:0019 - coimmunoprecipitation
P15499	Q9JJZ9	20238003	MI:0019 - coimmunoprecipitation
P16054	Q91YM2	14636894	MI:0019 - coimmunoprecipitation
P17679	Q80YQ2	17132730	MI:0096 - pull down
P17679	Q924H2	17132730	MI:0096 - pull down
P17679	Q9CQA5	17132730	MI:0096 - pull down
P17679	Q9CZB6	17132730	MI:0096 - pull down
P17679	Q9D7W5	17132730	MI:0096 - pull down
P17918	Q9ESU6	12192049	MI:0006 - anti bait coimmunoprecipitation
P20917	P11276	2446864	MI:0892 - solid phase assay
P20917	P13595	2446864	MI:0892 - solid phase assay
P20917	P20917	2446864	MI:0892 - solid phase assay
P20917	Q8K0S5	17189258	MI:0415 - enzymatic studies
P21183	P04401	1942734	MI:0004 - affinity chromatography technologies
P23804	P02340	10075719	MI:0019 - coimmunoprecipitation
P26450	P97465	18204460	MI:0019 - coimmunoprecipitation
P26955	P04351	1695379	MI:0027 - cosedimentation
P26955	P04401	8258700	MI:0004 - affinity chromatography technologies
P26955	P07321	1695379	MI:0027 - cosedimentation
P26955	P15247	1695379	MI:0027 - cosedimentation
P27601	Q76MZ3	15525651	MI:0059 - gst pull down
P27699	Q8R4I4	14512522	MI:0018 - two hybrid
P28651	Q9ERP3	18157088	MI:0018 - two hybrid
P29351	Q8C180	9632781	MI:0019 - coimmunoprecipitation
P30658	A2AM29	11439343	MI:0018 - two hybrid
P30658	P30658	9858600	MI:0018 - two hybrid
P31786	O89090	14530276	MI:0040 - electron microscopy
P35279	Q91V27	11856727	MI:0007 - anti tag coimmunoprecipitation
P35285	Q80T23	12051743	MI:0096 - pull down
P35285	Q91V27	11856727	MI:0007 - anti tag coimmunoprecipitation
P35293	Q80T23	12051743	MI:0096 - pull down
P35293	Q91V27	11856727	MI:0007 - anti tag coimmunoprecipitation
P35295	Q80T23	12051743	MI:0096 - pull down
P35295	Q91V27	11856727	MI:0007 - anti tag coimmunoprecipitation
P35396	Q6PDY0	15644333	MI:0007 - anti tag coimmunoprecipitation
P35917	Q00731	7970715	MI:0004 - affinity chromatography technologies
P37889	Q9QZJ6	17324935	MI:0892 - solid phase assay
P39447	Q8VHG2	16043488	MI:0019 - coimmunoprecipitation
P41593	Q9WTJ5	15823033	MI:0019 - coimmunoprecipitation, MI:0113 - western blot
P42586	Q62414	14573534	MI:0019 - coimmunoprecipitation
P43407	P21981	20929862	MI:0019 - coimmunoprecipitation
P46414	Q03147	7954814	MI:0019 - coimmunoprecipitation
P46662	Q61391	19388049	MI:0019 - coimmunoprecipitation
P47708	P61027	11773082	MI:0007 - anti tag coimmunoprecipitation
P51141	Q9WV60	10428961	MI:0007 - anti tag coimmunoprecipitation
P51150	Q80T23	12051743	MI:0096 - pull down
P51150	Q91V27	11856727	MI:0007 - anti tag coimmunoprecipitation
P51680	P10855	9192769	MI:0405 - competition binding
P51680	P14097	9192769	MI:0405 - competition binding
P51680	P30882	9192769	MI:0405 - competition binding
P53566	Q6PDY0	15644333	MI:0007 - anti tag coimmunoprecipitation
P54130	P16092	8619928	MI:0004 - affinity chromatography technologies
P54130	Q03142	8619928	MI:0004 - affinity chromatography technologies
P55258	Q80T23	12051743	MI:0096 - pull down
P55258	Q91V27	11856727	MI:0007 - anti tag coimmunoprecipitation
P55258	Q99N48	11773082	MI:0007 - anti tag coimmunoprecipitation
P55258	Q99N50	11773082	MI:0007 - anti tag coimmunoprecipitation
P55258	Q9R0Q1	11773082	MI:0007 - anti tag coimmunoprecipitation
P56371	Q80T23	12051743	MI:0096 - pull down
P56371	Q91V27	11856727	MI:0007 - anti tag coimmunoprecipitation
P56371	Q9D0C1	12972561	MI:0096 - pull down; MI:0019
P56546	P30658	9858600	MI:0018 - two hybrid
P61027	Q80T23	12051743	MI:0096 - pull down
P61027	Q91V27	11856727	MI:0007 - anti tag coimmunoprecipitation
P61027	Q99N48	11773082	MI:0007 - anti tag coimmunoprecipitation
P61027	Q99N50	11773082	MI:0007 - anti tag coimmunoprecipitation
P61027	Q9R0Q1	11773082	MI:0007 - anti tag coimmunoprecipitation
P61588	P70336	16472685	MI:0217 - phosphorylation reaction
P62259	Q61097	10891492	MI:0019 - coimmunoprecipitation
P62492	Q80T23	12051743	MI:0096 - pull down
P62492	Q91V27	11856727	MI:0007 - anti tag coimmunoprecipitation

P62500	Q00322	15644333	MI:0007 - anti tag coimmunoprecipitation
P62821	Q80T23	12051743	MI:0096 - pull down
P62821	Q91V27	11856727	MI:0007 - anti tag coimmunoprecipitation
P62880	P63213	1629181	MI:0004 - affinity chromatography technologies
P63011	Q91V27	11856727	MI:0007 - anti tag coimmunoprecipitation
P63011	Q99N48	11773082	MI:0007 - anti tag coimmunoprecipitation
P63011	Q99N50	11773082	MI:0007 - anti tag coimmunoprecipitation
P63011	Q9R0Q1	11773082	MI:0007 - anti tag coimmunoprecipitation
P63101	Q61097	10891492	MI:0019 - coimmunoprecipitation
P63239	Q58A65	17074887	MI:0018 - two hybrid
P63330	Q9EP53	18291711	MI:0019 - coimmunoprecipitation
P70196	Q62523	17092936	MI:0007 - anti tag coimmunoprecipitation; MI:0096
P70196	Q8BFW7	17092936	MI:0007 - anti tag coimmunoprecipitation; MI:0096
P70217	P97471	16087734	MI:0018 - two hybrid
P70217	Q60954	15617687	MI:0018 - two hybrid
P70371	Q3UES3	12080061	MI:0004 - affinity chromatography technologies
P70371	Q6PFX9	12080061	MI:0004 - affinity chromatography technologies
P70452	Q549X6	16186111	MI:0007 - anti tag coimmunoprecipitation
P97428	Q9Z0G0	9770488	MI:0018 - two hybrid
P97503	Q8C2B3	14612411	MI:0006 - anti bait coimmunoprecipitation
P97503	Q9WU42	14612411	MI:0006 - anti bait coimmunoprecipitation
Q01279	Q9QUQ5	16144838	MI:0006 - anti bait coimmunoprecipitation
Q02248	Q02257	17925400	MI:0006 - anti bait coimmunoprecipitation
Q02248	Q8BN78	10207085	MI:0019 - coimmunoprecipitation
Q04863	Q04207	9070378	MI:0004 - affinity chromatography technologies
Q08879	Q9QZJ6	17324935	MI:0892 - solid phase assay
Q09163	Q01705	20457810	MI:0019 - coimmunoprecipitation
Q505F1	Q60953	19204783	MI:0019 - coimmunoprecipitation
Q548T0	Q549X6	16186111	MI:0007 - anti tag coimmunoprecipitation
Q548T0	Q80T23	16186111	MI:0007 - anti tag coimmunoprecipitation
Q548T0	Q99N48	16186111	MI:0007 - anti tag coimmunoprecipitation
Q548T0	Q99N50	16186111	MI:0007 - anti tag coimmunoprecipitation
Q549X6	Q60770	16186111	MI:0007 - anti tag coimmunoprecipitation
Q60770	Q8K1E0	16186111	MI:0007 - anti tag coimmunoprecipitation
Q60972	Q01147	10866654	MI:0018 - two hybrid
Q61146	Q8VHG2	16043488	MI:0019 - coimmunoprecipitation
Q61205	Q924X6	17330141	MI:0019 - coimmunoprecipitation
Q61206	Q924X6	17330141	MI:0019 - coimmunoprecipitation
Q61559	P27512	21148035	MI:0110 - text mining, MI:0114 x-ray crystallography
Q61730	P01582	17669273	MI:0030 - cross-linking study
Q62009	Q08024	9632804	MI:0004 - affinity chromatography technologies
Q62108	P28652	19455133	MI:0676 - tandem affinity purification
Q62312	Q8CIN4	14612425	MI:0006 - anti bait coimmunoprecipitation
Q62424	P97471	16087734	MI:0018 - two hybrid
Q63912	Q7M6Z0	17189258	MI:0415 - enzymatic studies
Q63912	Q8K0S5	17189258	MI:0415 - enzymatic studies
Q64092	Q03141	20214879	MI:0019 - coimmunoprecipitation
Q64343	Q9DBM0	14504269	MI:0019 - coimmunoprecipitation
Q64729	Q8CIN4	14612425	MI:0006 - anti bait coimmunoprecipitation
Q66T02	P60766	20811643	MI:0096 - pull down
Q6NXK8	Q62108	19571134	MI:0019 - coimmunoprecipitation
Q6TDP3	Q9WTX6	17062563	MI:0019 - coimmunoprecipitation
Q6Y5D8	Q8CIN4	115471851	MI:0018 - two hybrid
Q71VB4	Q7TPV4	17875935	MI:0019 - coimmunoprecipitation
Q7M6Z0	Q8K0T0	17189258	MI:0415 - enzymatic studies
Q7M6Z0	Q99P72	17189258	MI:0415 - enzymatic studies
Q7M6Z0	Q9ES97	17189258	MI:0415 - enzymatic studies
Q7TMS5	Q9DBM0	14504269	MI:0019 - coimmunoprecipitation
Q80T23	Q99KL7	12051743	MI:0096 - pull down
Q80T23	Q9CQD1	12051743	MI:0096 - pull down
Q80T23	Q9R0M6	12051743	MI:0096 - pull down
Q80T23	Q9WTL2	12051743	MI:0096 - pull down
Q8BGS1	P62331	18794329	MI:1016 - fluorescence recovery after photobleaching
Q8BPB5	Q9QZJ6	17324935	MI:0892 - solid phase assay
Q8CBD1	P68254	18628823	MI:0019 - coimmunoprecipitation
Q8CBD1	Q9JIF0	18628823	MI:0019 - coimmunoprecipitation, MI:0110 - text mining
Q8CF89	Q62312	19556242	MI:0019 - coimmunoprecipitation
Q8CF89	Q64729	19556242	MI:0019 - coimmunoprecipitation
Q8CIN4	Q8CIN4	115471851	MI:0018 - two hybrid
Q8K0S5	Q8K0T0	17189258	MI:0415 - enzymatic studies
Q8K0S5	Q99P72	17189258	MI:0415 - enzymatic studies

Q8K0S5	Q9ES97	17189258	MI:0415 - enzymatic studies
Q8K0T0	Q99PI8	17189258	MI:0415 - enzymatic studies
Q8R4G0	O08747	14595443	MI:0004 - affinity chromatography technologies
Q8R4G0	Q8K1S3	14595443	MI:0004 - affinity chromatography technologies
Q8R4G0	Q8K1S4	14595443	MI:0004 - affinity chromatography technologies
Q8VHZ5	Q9EP53	19129461	MI:0019 - coimmunoprecipitation
Q91V27	Q99KL7	11856727	MI:0007 - anti tag coimmunoprecipitation
Q91V27	Q9CQD1	11856727	MI:0007 - anti tag coimmunoprecipitation
Q91V27	Q9R0M6	11856727	MI:0007 - anti tag coimmunoprecipitation
Q91V27	Q9WTL2	11856727	MI:0007 - anti tag coimmunoprecipitation
Q922H7	O35182	15239668	MI:0019 - coimmunoprecipitation
Q922H7	O35253	15239668	MI:0019 - coimmunoprecipitation
Q922H7	P97471	15239668	MI:0019 - coimmunoprecipitation
Q922H7	Q62432	15239668	MI:0019 - coimmunoprecipitation
Q922H7	Q8BUN5	15239668	MI:0019 - coimmunoprecipitation
Q99K43	P38532	18570919	MI:0018 - two hybrid
Q9EQW6	Q99MA9	14573534	MI:0019 - coimmunoprecipitation
Q9ERI2	Q99104	12006666	MI:0004 - affinity chromatography technologies
Q9ERP3	Q9JK37	18157088	MI:0018 - two hybrid
Q9JM90	Q9QVP9	10679268	MI:0018 - two hybrid
Q9JMB0	P05132	10671526	MI:0018 - two hybrid
Q9JMB0	P0C605	10671526	MI:0018 - two hybrid
Q9QXX8	Q61584	10556305	MI:0018 - two-hybrid, MI:0059 - gst pull down
Q9QZJ6	Q9WVH9	17324935	MI:0892 - solid phase assay
Q9QZJ6	Q9WVJ9	17324935	MI:0892 - solid phase assay
Q9WTW2	P23299	19623261	MI:0019 - coimmunoprecipitation
O08599	P70452	16186111	MI:0007 - anti tag coimmunoprecipitation

Table S3. CORUM database complexes from mouse (Mus musculus).

**Parvulin-associated pre-rRNP** complex ID: 3047 PubMed ID: 11960984		**Respiratory chain complex I** complex ID: 382 PubMed ID: 15591592		**20S Immuno-proteasome** complex ID: 39 PubMed ID: 10436176		**c-Src immune complex** complex ID: 6938 PubMed ID: 19628583		**DNA synthesome complex** complex ID: 1001 PubMed ID: 8126085	
**Protein**	**Name**	**Protein**	**Name**	**Protein**	**Name**	**Protein**	**Name**	**Protein**	**Name**
O09167	Rpl21	O09111	Ndufb11	O09061	Psmb1	P05213	Tuba1b	O35654	Pold2
O43143	DHX15	O35683	Ndufa1	O35955	Psmb10	P05480	Src	P17918	Pcna
O76021	RSL1D1	P03888	Mtnd1	O70435	Psma3	P16054	Prkce	P20664	Prim1
O95995	GAS8	P03899	Mtnd3	P28063	Psmb8	P20444	Prkca	P33609	Pola1
P09405	Ncl	P03911	Mtnd4	P28076	Psmb9	P28867	Prkcd	P33610	Prim2
P11276	Fn1	P52503	Ndufs6	P49722	Psma2	P34152	Ptk2	P33611	Pola2
P12970	Rpl7a	Q3UIU2	Ndufb6	P99026	Psmb4	P49817	Cav1	P37913	Lig1
P14148	Rpl7	Q62425	Ndufa4	Q9QUM9	Psma6	P63085	Mapk1	P52431	Pold1
P14869	Rplp0	Q7TMF3	Ndufa12	Q9R1P0	Psma4	P68369	Tuba1a	Q01320	Top2a
P19253	Rpl13a	Q8K3J1	Ndufs8	Q9R1P1	Psmb3	Q01776	Gnrhr	Q04750	Top1
P21127	CDK11B	Q91VD9	Ndufs1	Q9R1P3	Psmb2	Q61097	Ksr1	Q9CWP8	Pold4
P27635	RPL10	Q91WD5	Ndufs2	Q9R1P4	Psma1	Q63844	Mapk3	Q9EQ28	Pold3
P27659	Rpl3	Q91WP8	Ndufv3	Q9Z2U0	Psma7	Q64727	Vcl		
P35550	Fbl	Q91YT0	Ndufv1	Q9Z2U1	Psma5	Q8VI36	Pxn		
P35980	Rpl18	Q99LC3	Ndufa10						
P46781	RPS9	Q99LY9	Ndufs5						
P47911	Rpl6	Q9CPP6	Ndufa5						
P47962	Rpl5	Q9CPU2	Ndufb2						
P47963	Rpl13	Q9CQ54	Ndufc2						
P53569	Cebpz	Q9CQ75	Ndufa2						
P56183	Rrp1	Q9CQ91	Ndufa3						
P61255	Rpl26	Q9CQC7	Ndufb4						
P62242	Rps8	Q9CQH3	Ndufb5						
P62702	Rps4x	Q9CQJ8	Ndufb9						
P62717	Rpl18a	Q9CQZ5	Ndufa6						
P62754	Rps6	Q9CQZ6	Ndufb3						
P62906	RPL10A	Q9CR21	Ndufab1						
P62908	Rps3	Q9CR61	Ndufb7						
P62918	Rpl8	Q9CXZ1	Ndufs4						
P68363	TUBA1B	Q9D6J5	Ndufb8						
P84099	Rpl19	Q9D6J6	Ndufv2						
P97351	Rps3a	Q9D8B4	Ndufa11						
P97452	Bop1	Q9DC69	Ndufa9						
P99024	Tubb5	Q9DC70	Ndufs7						
Q61656	Ddx5	Q9DCJ5	Ndufa8						
Q6DFW4	Nop58	Q9DCS9	Ndufb10						
Q7TPV4	Mybbp1a	Q9DCT2	Ndufs3						
Q8K363	Ddx18	Q9ERS2	Ndufa13						
Q91VE6	Nifk	Q9MD82	mt-Nd5						
Q921N6	Ddx27	Q9Z1P6	Ndufa7						
Q922K7	Nop2								
Q99LH1	Gnl2								
Q99ME9	Gtpbp4								
Q9BWT6	MND1								
Q9CR57	Rpl14								
Q9CYH6	Rrs1								
Q9CZM2	Rpl15								
Q9D0I8	Mrto4								
Q9D0R4	Ddx56								
Q9D6Z1	Nop56								
Q9D8E6	Rpl4								
Q9D8N0	Eef1g								
Q9D903	Ebna1bp2								
Q9DBE9	Ftsj3								
Q9EQ61	Pes1								
Q9ESV0	Ddx24								
Q9JIK5	Ddx21								
Q9JJ80	Rpf2								
Q9JJA4	Wdr12								
Q9NW13	RBM28								
Q9ULW0	TPX2								
Q9Y237	PIN4								

Table S4. Proteins identified to be localized to lipid rafts upon B cell activation.

Proteins 1-65		Proteins 66-130		Proteins 131-195		Proteins196-260		Proteins 261-324	
UniProt	Name	UniProt	Name	UniProt	Name	UniProt	Name	UniProt	Name
Q8BGQ7	Aars	Q9WVK4	Ehd1	Q8R081	Hnrnpl	Q9ERS5	Plekha2	P11157	Rrm2
Q99LE6	Abcf2	Q8JZQ9	Eif3b	P07901	Hsp90aa1	Q9D3P8	Plgrkt	Q99LF4	Rtcb
Q8K268	Abcf3	Q8R1B4	Eif3c	P20029	Hspa5	Q923G2	Polr2h	Q99P72	Rtn4
Q91V92	Acly	P60229	Eif3e	P63017	Hspa8	Q91YU8	Ppan	P60122	Ruvbl1
Q9D358	Acp1	Q8QZY1	Eif3l	Q61699	Hsph1	P62137	Ppp1ca	Q3UU41	Scimp
Q9QUJ7	Acsl4	Q99JX4	Eif3m	Q6ZQA6	Igsf3	P63330	Ppp2ca	O08547	Sec22b
P60710	Actb	P60843	Eif4a1	O54824	Il16	P35700	Prdx1	Q9CY58	Serbp1
P63260	Actg1	Q8BGD9	Eif4b	P24547	Impdh2	Q61171	Prdx2	Q8VIJ6	Sfpq
P57780	Actn4	Q6NZJ6	Eif4g1	Q9EPL8	Ipo7	O08807	Prdx4	Q9ET39	Slamf6
P61161	Actr2	Q80XI3	Eif4g3	Q9JKF1	Iqgap1	P68404	Prkcb	P53986	Slc16a1
Q8BK64	Ahsa1	P63242	Eif5a	P81122	Irs2	Q8CIG8	Prmt5	Q8JZU2	Slc25a1
O08915	Aip	P70372	Elavl1	Q9JHU9	Isyna1	Q9D7G0	Prps1	Q8BH59	Slc25a12
P05064	Aldoa	Q8BPU7	Elmo1	O54890	Itgb3	Q8BHE0	Prr11	Q9Z2Z6	Slc25a20
P97384	Anxa11	P21995	Emb	Q3U0V1	Khsrp	P62192	Psmc1	Q8VEM8	Slc25a3
Q07076	Anxa7	P17182	Eno1b	Q61768	Kif5b	O88685	Psmc3	P48962	Slc25a4
P84078	Arf1	P48193	Epb4.1	P52293	Kpna2	P62196	Psmc5	P10852	Slc3a2
P61750	Arf4	O70318	Epb4.1l2	P70168	Kpnb1	P62334	Psmc6	Q99JR8	Smarcd2
P62331	Arf6	Q8BGS1	Epb4.1l5	Q61595	Ktn1	Q8BG32	Psmd11	Q8CG48	Smc2
Q3UIA2	Arhgap17	Q8CGC7	Eprs	Q8BMJ2	Lars	Q99JI4	Psmd6	Q9CW03	Smc3
Q8VDN2	Atp1a1	P42567	Eps15	Q3U9G9	Lbr	P29351	Ptpn6	P62320	Snrpd3
O55143	Atp2a2	Q61545	Ewsr1	Q61233	Lcp1	O35295	Purb	Q3UMC0	Spata5
Q9Z1G3	Atp6v1c1	P26040	Ezr	Q99N69	Lpxn	Q8BML9	Qars	Q9CYN2	Spcs2
P28658	Atxn10	Q6A0A9	Fam120a	P19973	Lsp1	Q91V41	Rab14	Q64337	Sqstm1
Q7TQH0	Atxn2l	P19096	Fasn	Q01965	Ly9	P35293	Rab18	Q6P069	Sri
B2RQC6	Cad	P35550	Fbl	Q8C052	Map1s	P62821	Rab1	Q60864	Stip1
P35564	Canx	P08101	Fcgr2b	Q68FL6	Mars	Q9D1G1	Rab1b	P46978	Stt3a
P47754	Capza2	Q8K1B8	Fermt3	Q8VCF0	Mavs	P35282	Rab21	Q6A028	Swap70
P47757	Capzb	Q69ZL1	Fgd6	Q9JKP5	Mbnl1	Q9CQD1	Rab5a	Q7TMK9	Syncrip
Q9WU84	Ccs	P30416	Fkbp4	P25206	Mcm3	P35278	Rab5c	Q9WVA4	Tagln2
P80315	Cct4	Q8BTM8	Flna	P49717	Mcm4	P35279	Rab6a	Q921F2	Tardbp
P42932	Cct8	Q80X90	Flnb	Q61881	Mcm7	P51150	Rab7	P10711	Tcea1
P25918	Cd19	P09528	Fth1	Q9CQT1	Mri1	P63001	Rac1	Q62351	Tfrc
Q61470	Cd37	Q91WJ8	Fubp1	P19437	Ms4a1	Q05144	Rac2	Q9D880	Timm50
P21855	Cd72	Q91Z49	Fyttd1	P26041	Msn	Q9WVM1	Racgap1	P40142	Tkt
P40237	Cd82	P97379	G3bp2	P00405	mt-Co2	Q9ERU9	Ranbp2	P26039	Tln1
P60766	Cdc42	Q9CZD3	Gars	Q922D8	Mthfd1	Q8BMS9	Rassf2	Q61029	Tmpo
P11440	Cdk1	Q64737	Gart	Q8R1S4	Mtss1	Q91VM5	Rbmxl1	Q8BFY9	Tnpo1
Q4VAA2	Cdv3	Q8VHR5	Gatad2b	Q7TPV4	Mybbp1a	Q8BK67	Rcc2	P17751	Tpi1
Q8BT07	Cep55	O09172	Gclm	Q99KQ4	Nampt	Q9WUK4	Rfc2	P21107	Tpm3
P18760	Cfl1	E9PVA8	Gcn1l1	Q8C156	Ncaph	Q6A0D4	Rftn1	Q64514	Tpp2
Q9CRB9	Chchd3	Q61598	Gdi2	P46935	Nedd4	Q9QUI0	Rhoa	P63028	Tpt1
Q9Z1Q5	Clic1	Q6Y7W8	Gigyf2	Q8K4Q6	Neil1	P84096	Rhog	P99024	Tubb5
Q9QYB1	Clic4	Q9WU65	Gk2	Q9WTK5	Nfkb2	Q9QZL0	Ripk3	P10639	Txn1
Q68FD5	Cltc	Q3THK7	Gmps	Q01768	Nme2	Q6ZWV3	Rpl10	Q9D883	U2af1
Q9DBP5	Cmpk1	P27601	Gna13	Q8K2T1	Nmral1	P84099	Rpl19	P0DP28	unknown
P53996	Cnbp	P08752	Gnai2	Q61937	Npm1	P62830	Rpl23	Q02053	Uba1
P16330	Cnp	Q9DC51	Gnai3	Q9R1J0	Nsdhl	P27659	Rpl3	Q91VX2	Ubap2
O88207	Col5a1	P63094	Gnas	Q5F2E7	Nufip2	P62889	Rpl30	Q80X50	Ubap2l
O89053	Coro1a	P62880	Gnb2	Q80U93	Nup214	Q9D8E6	Rpl4	Q9EPU0	Upf1
Q8CJ40	Crocc	Q9QYE6	Golga5	Q8BJ71	Nup93	P47911	Rpl6	P52479	Usp10
P70698	Ctps	Q8BUV3	Gphn	Q7TQI3	Otub1	P14148	Rpl7	Q3UJD6	Usp19
Q922B2	Dars	O70325	Gpx4	B2RRE7	Otud4	Q91YQ5	Rpn1	Q9Z1Q9	Vars
Q91VR5	Ddx1	Q99L20	Gstt3	P29341	Pabpc1	P63325	Rps10	Q01853	Vcp
Q501J6	Ddx17	P53702	Hccs	Q9DCL9	Paics	P62245	Rps15a	Q60930	Vdac2
Q62167	Ddx3x	P49710	Hcls1	Q3TC46	Patl1	P14131	Rps16	Q60931	Vdac3
P54823	Ddx6	Q9JKY5	Hip1r	P60335	Pcbp1	P63276	Rps17	P62960	Ybx1
P00375	Dhfr	Q00547	Hmmr	Q9WU78	Pdcd6ip	P60867	Rps20	P59326	Ythdf1
Q811D0	Dlg1	O70252	Hmox2	Q922R8	Pdia6	P62849	Rps24	Q91YT7	Ythdf2
P63037	Dnaja1	P49312	Hnrnpa1	Q8R1G6	Pdlim2	Q6ZWU9	Rps27rt	Q9CQV8	Ywhab
Q9QYJ0	Dnaja2	O88569	Hnrnpa2b1	Q9WUA3	Pfkp	Q6ZWY3	Rps27l	P62259	Ywhae
Q8C147	Dock8	Q8BG05	Hnrnpa3	P62962	Pfn1	P97461	Rps5	P68510	Ywhah
Q9JHU4	Dync1h1	Q99020	Hnrnpab	Q9JJV2	Pfn2	P62754	Rps6	P63101	Ywhaz
P57776	Eef1d	Q9Z2X1	Hnrnpf	Q61753	Phgdh	P14206	Rpsa	Q3UPF5	Zc3hav1
Q9D8N0	Eef1g	O35737	Hnrnph1	Q7M6Y3	Picalm	P62071	Rras2	B2RX14	Zcchc11
P58252	Eef2	P61979	Hnrnpk	Q8CIH5	Plcg2	P07742	Rrm1		

Table S5. Proteins predicted to coevolve with Cad.

**UniProt ID**	**Name**	**Residues coevolving with Cad**	**Subcellular location**
Q8JZQ9	Eif3b	207I	Cytoplasm
Q8CGC7	Eprs	5C, 12N, 747A	Cytoplasm, Membrane
P19096	Fasn	2268Q	Cytoplasm; GO: Golgi, Mitochondrion, membrane
P19437	Ms4a1	191M, 213M, 219S	Membrane (multipass); GO: Nucleoplasm
Q80U93	Nup214	1513A, 1511P	Nuclear pore (cytoplasmic side)
Q9ERU9	Ranbp2	229R	Nucleus, Nuclear pore, Nuclear envelope; GO: mitochondrion
Q91YQ5	Rpn1	89I, 86G, 381N, 389D, 491L	ER; GO: Cytosol,
Q99P72	Rtn4	248T, 263R, 266N, 309V, 324H	Membrane, ER; GO: Nuclear envelope
P10852	Slc3a2	165I	Membrane, Lysosome membrane; GO: nucleoplasm
Q3UMC0	Spata5	507A, 538R, 589S, 609I	Mitochondrion, Cytoplasm, Spindle
**UniProt**	**Name**	**Residues coevolving from Cad**	**Subcellular location**
B2RQC6	**Cad**	20A, 1728L, 1887G, 1892A, 2108S, 2114S, 2610A	Cytoplasm, Nucleus
